# A low-cost "plant-scanner" platform for automated detection of *Ustilago maydis* infection in maize using deep learning

**DOI:** 10.1038/s41598-026-60714-4

**Published:** 2026-07-11

**Authors:** Marvin Christ, Seyed Amir Hossein Tabatabaei, Niklas Ostwald, Oskar Seifert, Itzel Rubio Elizalde, Paul Klemm, Clemens Thölken, Marcus Lechner, Nasim Faridnia, Gert Bange, Keywan Sohrabi

**Affiliations:** 1 Center for Synthetic Microbiology (SYNMIKRO) and Department of Chemistry, University of Marburg, Karl-von-Frisch Strasse 14, 35043 Marburg, Germany; 2 Department of Computer Science, Faculty of Mathematical Sciences, University of Guilan, Namjoo St., Rasht, 41938-33697 Iran; 3 Faculty of Health Sciences, University of Applied Sciences (Technische Hochschule Mittelhessen), Wiesenstrasse 14, 35390 Giessen, Germany; 4 Department of Computer Science, University of Texas, San Antonio, USA; 5 Max-Planck-Institute for Terrestrial Microbiology, Karl-von-Frisch Strasse 14, 35043 Marburg, Germany

**Keywords:** *Ustilago maydis*, Machine learning, Platform, Deep learning, YOLO, Transfer learning, Infection, Computational biology and bioinformatics, Engineering, Mathematics and computing, Plant sciences

## Abstract

*Ustilago maydis* is a biotrophic fungus that causes smut disease in maize, leading to tumor formation on aerial parts of the plant. While *U. maydis* has been a model for plant-fungal interaction studies, no tool has existed to automatically quantify infection symptoms under laboratory conditions for deep learning analysis. To address this, we developed a rotating camera system that captures videos of plants under customized lighting and shutter settings. These videos were used to train machine learning models to distinguish between healthy and infected plants. Two detection approaches have been presented. In the first approach, by employing a naive masking technique and combining classical machine learning classifiers utilizing handcrafted features, the model achieved a reasonable performance, with an Area Under the Curve (AUC) of maximum 0.90 on the Receiver Operating Characteristic in one of the classifiers, showing relatively high sensitivity and specificity. The second approach utilizes pre-trained YOLO11 model for object detection and further classification. The YOLO11-based approach outperforms traditional methods, achieving near-perfect validation accuracy (AUC: 0.99–1.00), demonstrating its superiority for real-time, scalable applications. Our toolset, featuring a cost-efficient and customizable scanning platform with open building-blocks design, provides a valuable resource as a proof-of-concept for unbiased disease symptom detection and scoring, with potential applications in other plant pathology studies. This point enables easy replication and adaptation by other research laboratories which makes the platform robust, scalable and practical beyond our specific application.

## Introduction

### The corn smut fungus *Ustilago maydis* as a model system to study biotrophic pathogens

**Fig. 1:**
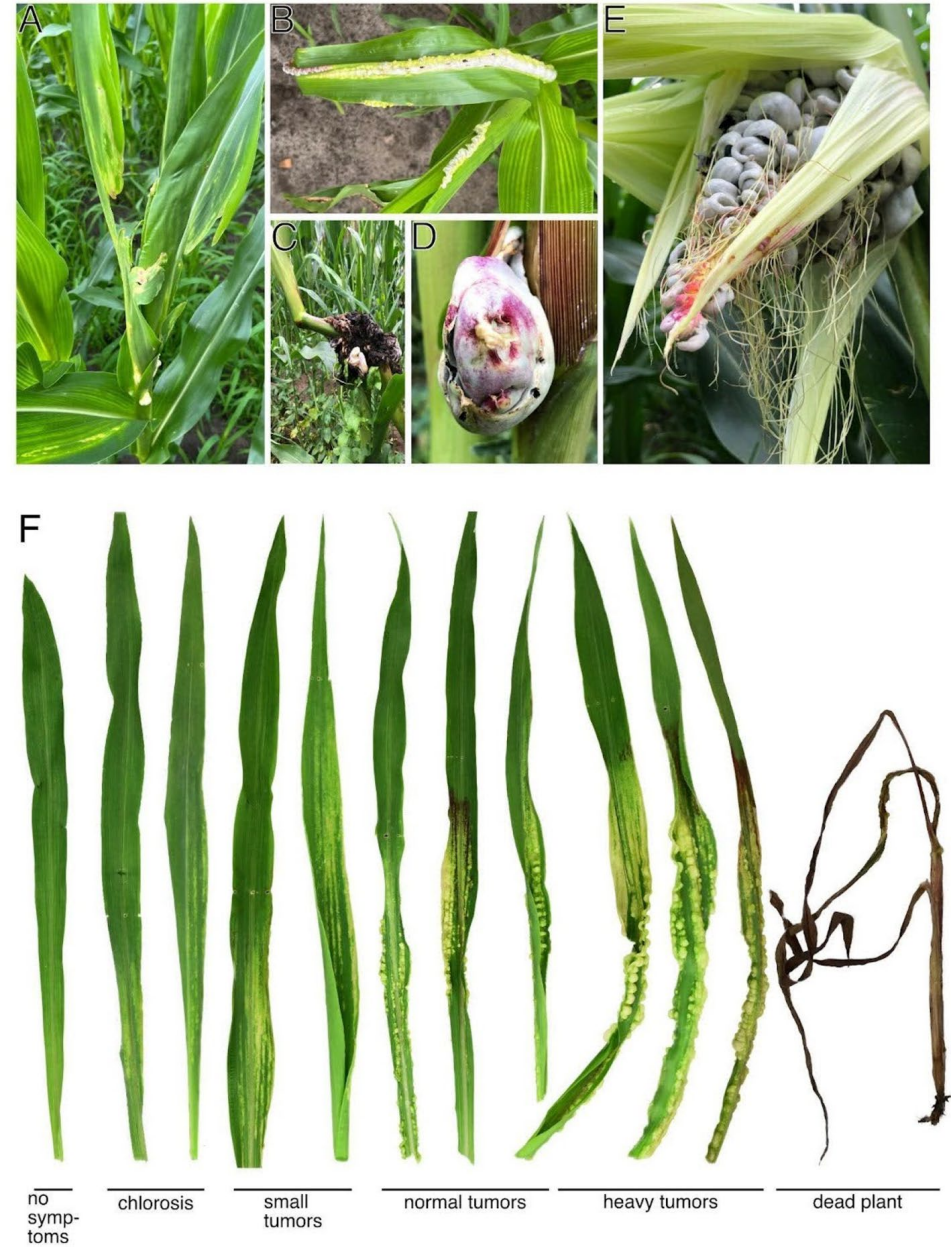
Infection symptoms of *U. maydis* on maize plants in the field and in the greenhouse. Panels (**A**–**E**) show field-cultivated maize plants with natural infection symptoms in increasing stages from chlorosis to spore-filled galls. The pictures were taken during summertime in corn fields in Germany. Panel (**F**) exemplifies the different infection symptoms in artificially infected maize plants cultivated under controlled conditions in a greenhouse.

Eradicating plant diseases presents a significant challenge in agriculture and global food security, as these cause an approximate annual loss of up to $40\%$ global crop production^1,2^. This problem is set to increase as temperatures rise, promoting the spread of pathogens such as fungi^3^. To address this problem, the study and understanding of plant-fungi interactions through model organisms is required for the development of accurate solutions. The biotrophic fungus *Ustilago maydis* has been included in the list of top ten fungal model organisms that have been established to study phytopathogen infection^4^. This is not particularly because of its economic importance, but because *U. maydis* is well-suited for genetics and molecular biology approaches, its genome is completely annotated and accessible for almost twenty years now^5^ and genetic manipulation via homologous recombination and/or CRISPR/Cas9 is well-established^6–8^. Furthermore, it is a representative of obligate biotrophic fungi which include smut, rust, and powdery mildew fungi. *U. maydis* specifically infects aerial parts of maize (*Zea mays*; Fig. 1A–E) and teosinte and triggers the formation of large tumors filled with diploid teliospores^9^). This pathosystem has been the subject of intense research for more than three decades attaining insight into different effector proteins secreted by *U. maydis* in order to manipulate the host plant (e.g.^10–12^ and how maize counteracts these challenges (e.g.^13,14^). Disease severity is often evaluated on young maize seedlings according to criteria developed by Kämper et al.^5^ (Fig. 1F). Infection symptoms are assessed by eye and categorized according to different criteria (Fig. 1F). This process is time-consuming, labor-intensive and prone to subjective or biased classification. An automated symptom-detection system combined with a deep learning model provides a valuable aid for members of the *U. maydis* research community and allows for comparable results in a uniform system. Recent works^15,16^ underscore the need for automated tools to study *U. maydis* effector proteins, which our platform addresses.

### Plant disease detection by RGB imaging

Imaging in the visible light spectrum enables the large-scale, cost-effective and uncomplicated detection of plant disease symptoms (RGB imaging)^17–20^. RGB images can be used to extract the specific features for a particular type of plant infection of interest thereby enabling classification. Major features for classification are color, shape (reflected by changes in volume) and texture (e.g. homogeneity)^21^. RGB imaging of infected plants can then be used for training algorithms for plant disease detection in systematic studies^22^. Any state of the art digital camera is sufficient for many approaches, although other sensors have proven useful depending on the approach^18,19,23^. Selection of a suitable sensor depends not only on the desired level of detail accuracy of the features but also on factors such as acquisition costs, usability, integration capability, environmental influences and the object to be examined. Hyperspectral imaging and RGB imaging have proven to be particularly suitable in the last 10 years, as can be seen from existing image databases of infected plants used for training algorithms for plant disease detection in systematic review^22^. In laboratory environments or plant facilities, static or swiveling camera systems fixed on a tripod construction are often used^24–26^. Cell phone apps for detecting plant infections have also been developed in this context. Images or videos taken with the cell phone camera are then analyzed on the device in real time or via a connected data center^27,28^. Commercial systems such as the "WIWAM Phenotyping System"^29^ or the "Photon Systems Instruments Plant Screen System"^30^ were developed by others. Drones have been used for larger agricultural areas, which stand out from other systems in particular due to their spatial flexibility^31^. There are also approaches with mobile ground robots^32^. Furthermore, there are also mobile rail systems with a frame on top that travels over different parts of the field, both for large and small agricultural areas^33,34^. However, such systems differ substantially in cost, degree of automation, illumination standardization, and suitability for controlled laboratory infection assays. Smartphone-based solutions are inexpensive and portable, yet they often lack standardization in image quality, viewing angle, working distance, and lighting conditions. Drone- and robot-based systems are well suited for large-scale field monitoring but are less appropriate for small plants under controlled laboratory settings especially for host–pathogen interaction studies in which subtle differences in disease symptom development need to be assessed reproducibly across large numbers of plants. Commercial high-throughput phenotyping platforms offer excellent standardization and throughput; however, their high cost and limited accessibility may restrict their use in smaller research groups or resource-constrained environments. The proposed Plant-Scanner is strategically positioned between these approaches. As a proof-of-concept prototype, it establishes a foundation for future early-stage commercial development by demonstrating that standardized multi-view RGB imaging under controlled illumination can be achieved with low-cost, modular, and readily available components. This combination renders it particularly suitable for controlled laboratory infection assays and small-scale phenotyping studies, where reproducibility and cost-efficiency are paramount, while also providing a basis for subsequent translation toward a more robust commercial platform.

### Plant disease detection methods based on the machine learning and pattern recognition

Automated detection and analysis of plant diseases and pest identification are mainly based on image processing techniques, computer vision, traditional machine learning algorithms, and deep learning. In image processing and computer vision, conventional image processing algorithms and/or manual design of features are used, with the occasional use of miscellaneous classifiers^35–37^. Methods based on machine learning often begin with image processing techniques to enhance images and extract relevant features. Common techniques include image segmentation, edge detection, and color space transformations. These preprocessing steps help in detecting and isolating the infected regions and extracting discriminative features for subsequent analysis. In such methods, different properties of plant diseases and pests are used. However, fine-tuning the system, including adjusting the light source and shooting angle to achieve uniform illumination, is essential-though it can be a challenging task^38^. Image processing-based methods detect and classify the infected parts of the leaves by applying pre-processing techniques followed by clustering and segmentation of the leaves. The features including three key types, namely, color, shape, and texture are extracted to feed the possible augmented classifier. Several traditional machine learning algorithms have been applied to infection detection on plant leaves. These include decision trees, support vector machines (SVM), k-nearest neighbors (KNN), and random forests. These algorithms can effectively classify infected and healthy leaf regions based on extracted features. The proposed work in^21^ presents a survey reviewing the literature associated with plant disease detection based on machine learning. According to this work, the methods detect and classify the diseases with regard to the extracted feature types and the utilized machine learning model. For example in^39^, an HSI color conversion followed by color slicing technique is used to extract regions of interest achieving an accuracy of 0.966^21,39^. Alternatively, other color features including YCbCr, HIS and CIELAB can be used to recognize disease spots^40^. The work presented in^41^ utilized image color analysis and k-means clustering technique to detect and identify some special rice disease spots and leaves. The authors of^42^ have engaged the naive statistics of a wide variety of color image features augmented by traditional classifiers like SVM, KNN, and RF to achieve an accuracy of about 0.9465. Alternative works using color features for disease area segmentation can be named as^41,43–45^. As another example, color, shape and texture features including geometrical and texture indicators like area, perimeter, contrast, entropy, etc., have been extracted from the color features of the image in^46^. The feature set has been augmented by an SVM classifier achieving the accuracy about 0.972. In the work presented in^47^, three rice leaf diseases have been recognized using a combination of all features including color, shape, and texture types. The SVM classifier has been applied on the image wherein some initial background removals were applied to achieve the accuracy of about 0.8857. Other works combining the features can be mentioned as^21,48–50^. All of these methods and similar ones may struggle with complex and high-dimensional feature spaces and may not generalize well to unseen data. Also, it might be very difficult, even unrealistic to expect the classical image processing algorithms to completely eliminate the impact of background and/or scene change on the detection and recognition results^38^. There is a lot of instability when collecting plant disease and pest images shed by the natural light^35^. The aforementioned concerns as well as the incapability of classical and traditional image processing methods in effective feature extraction motivate the domain experts to elaborate the feature engineering part in disease detection and classification pipeline by the use of deep learning. Deep learning methods, particularly convolutional neural networks (CNNs), have shown remarkable success in various image analysis tasks, including infection detection on plant leaves. They have been applied to detect and classify the region of interest (infected parts) throughout two sequential phases consisting of the feature map generation and the classification followed. CNNs can automatically learn hierarchical representations from raw leaf images, eliminating the need for handcrafted features extraction. Transfer learning, where pre-trained models are fine-tuned on plant leaf datasets, has also proven effective in cases with limited training data. Using models from the CNN-based architecture YOLO (you only look once)^51^ has been shown to have promising results. Works recently presented in^52–54^ are some examples.

A survey is given in^55^ wherein the leaf disease detection methods based on deep learning and traditional machine learning as well as the challenges, limitations and applications are discussed. Similar survey has been presented in^56^ in which some recommendations to the farmers as well as researchers have been given as well. The presented survey in^57^ analyses the plant disease detection works based on traditional image processing and deep learning approaches as well. The methods are not limited to the individual ones stated as above and the hybrid methods which combine the aforementioned techniques are used as well. For example, machine learning algorithms, such as SVM and deep learning models, can be applied to the hyperspectral image data which captures spectral information at multiple wavelengths to identify spectral traces associated with infections. As another example, proposed framework in^58^ incorporates several different hybrid deep learning models containing pre-trained models to detect tomato early blight disease. The proposed framework performs extraordinary performance and achieves the accuracy in the range of 87.55 up to 1.00.

Although the shortlisted methods are utilized in order to detect the leaf disease in several different plants, there are some works which have been dedicated to detect the diseases appearing on maize leaves^59,60^. The proposed work in^59^ utilizes traditional machine learning algorithms to detect the maize leaf diseases based on the plant leaf images. The highest accuracy has been achieved via random forest classifier. The work presented in^60^ utilized the architecture of some pre-trained models for detection of three different leaf disease symptoms in maize plants. Also, they have embedded the detection framework in a mobile application for ease of use. With the emergence of attention mechanisms and transformer architectures, many plant disease detection and classification schemes have been developed upon these baselines. For example, the presented works in^61,62^ use Vision Transformers (ViTs) in detecting diseases in plant images captured in natural environments. The presented work in^63^ enhances interpretability in detecting and classifying leaf disease based on ViTs. The work presented in^64^ uses ensemble technique in integrating several deep learning architectures and transformers to identify and classify leaf diseases. With the combination of CNN and transformers, the proposed work in^65^ is able to detect the plant, leaf disease and well as severity of disease. Plant disease detection in Internet of Things (IoT) has been proposed based on similar combination approach in^66^. For example, the work presented in^67^ proposes a lightweight computer-vision framework for tomato leaf disease identification, aimed at practical deployment on mobile or portable devices. It combines data augmentation and background subtraction to isolate leaf regions, then uses a MobileNetV2-based classifier tuned by a modified Gray Wolf Optimization method. The resulting system is claimed to be highly accurate, and stable, while competitive with several standard deep learning baselines.

## Materials and methods

### Plant-sample generation

One to five seeds of the *Zea mays* cultivar ‘Early Golden Bantam’ (Urban Farmer) were planted 5–10 cm under the surface in 14 cm diameter and 11.5 cm high pots filled with peat moss (Heinrichs Agrar). Plants were grown in a plant chamber with $60\%$ humidity, day/night temperatures of $28^\circ / 20$ °C , and 14 hs photoperiod, with watering as needed. Under these conditions, after 7–8 days of growth, the plants reach the three-leaf stage and a height of about 15 cm. *Ustilago maydis* strains were grown in YEPSlight ($1\%$ w/v yeast extract, $0.4\%$ w/v peptone, and $0.4\%$ w/v sucrose) with a starting OD$_{600}$ = 0.2. Upon reaching OD$_{600}$ = 0.6–0.8 the cells were harvested and resuspended in sterile water to OD$_{600}$ = 1.500 µL were syringe-infected into the stems of the maize seedlings^5^. The videos of the plants were made over a period of up to 12 days post infection at various time points.

#### Video-sample generation of plants

During the development of the hardware, adjustments were continuously made to improve the quality of the individual raw images for better algorithm results. As listed here, this results in four different development phases of the scanner platform. In the following, we describe the development and improvement of the scanner, i.e., the intermediate stages of the project. Detailed specifications are mentioned in Subsect. The final plant scanning platform.

#### Phase one

Like in the PlantVillage^68^ database, we started to take pictures of infected plants with private cell phones. Recording parameters such as angles and distances were not uniformly chosen. The sodium vapor lamps in the plant chamber were the only light source, which made the images appear yellowish. This had a detrimental effect on the recognition of the fungal symptoms on the plant since some of them are naturally yellowish. Next, a small scanning device was designed in which a conventional cell phone was placed horizontally to the plant. In this setup, the camera was fixed, and the plant rotated around the camera. The rotation and vibrations caused exposed parts of the plant to shake, resulting in blurred video footage (no significant amount of video material was obtained).

#### Phase two

**Fig. 2:**
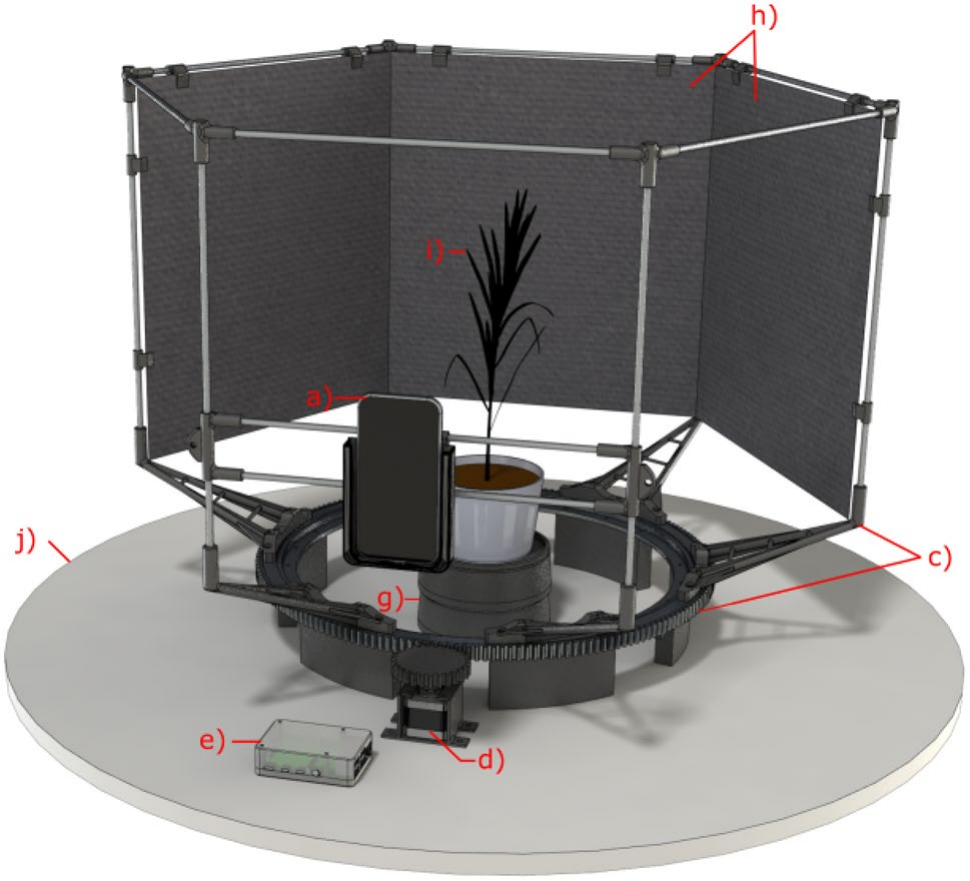
Phase Two Plantscanner for 360$^{\circ }$ image capturing of the infected plant with 150 L scan volume: (**a**) side-view camera (iPhone XRMobilephone), (**c**) 360$^{\circ }$ camera rotation unit, (**d**) stepper motor, (**e**) control unit (Arduino), (**g**) adjustable plant platform, (**h**) high-contrast background (black cardboard), (**i**) scan object, (**j**) base plate.

For phase 2 (Fig. 2), a platform was developed that uses a designated cell phone as a camera to move 360$^{\circ }$ around the plant to capture sharp images of the plant standing as still as possible. The ball-bearing rotary unit was driven by a stepper motor and an Arduino so that the desired rotational speed could be achieved. In order to achieve a constant light setting and thus remove unwanted background light, a conventional black cardboard paper was glued to the frame of the scanner as background. An adjustable plant platform was developed to position the plant to be scanned at the optimum height. The camera suspension could also be adjusted in height.

#### Phase three

**Fig. 3:**
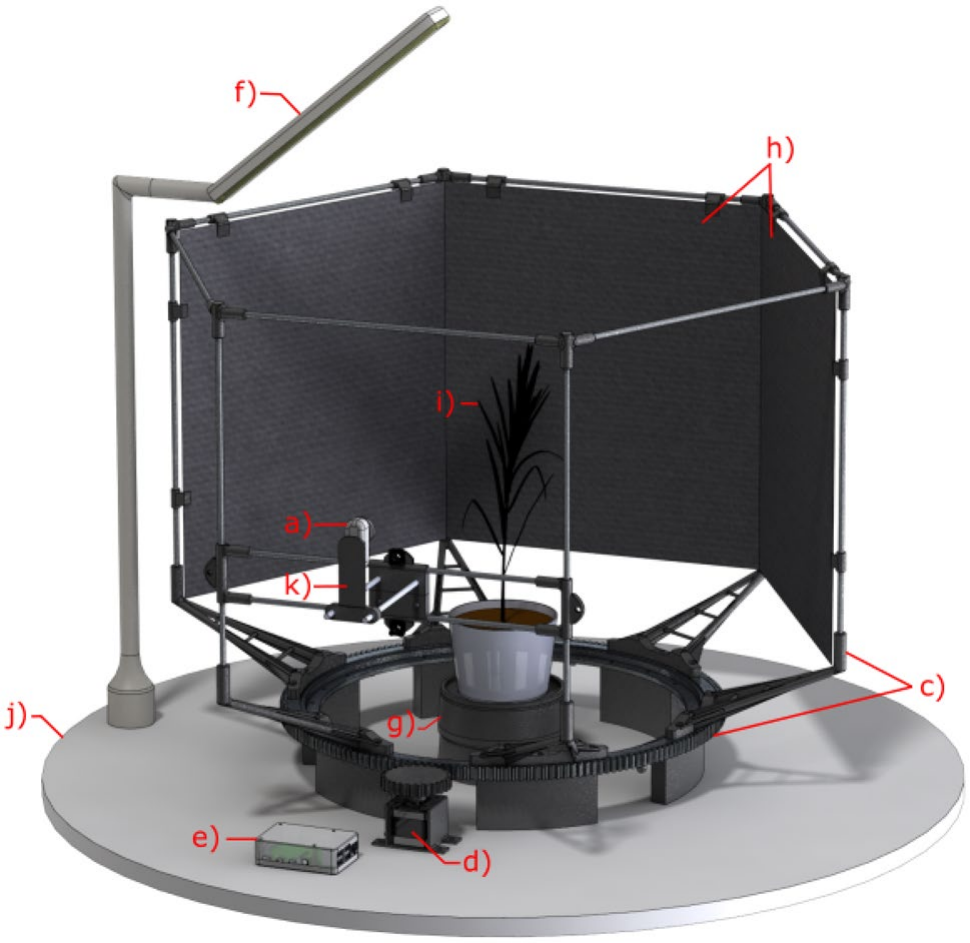
Phase Three Plantscanner for $360^{\circ }$ image capturing of the infected plant with 150 L scan volume: (**a**) side-view camera (Insta360 Go 2), (**c**) $360^{\circ }$ camera rotation unit, (**d**) stepper motor, (**e**) control unit, (**f**) lamp, (**g**) adjustable plant platform, (**h**) high-contrast background, (**i**) scan object, (**j**) base plate, (**k**) adjustable camera rail.

In phase 3, we opted for an Insta360 Go 2 action camera, which is suitable for capturing images in motion/vibration. We also increased the distance between the camera and the plant to capture entire plants at an advanced stage of growth. In addition, we installed an LED light source with adjustable light intensity to generate a consistent light environment. The description is depicted in Fig. 3.

#### Phase four

Phase four was the final development phase and served as a validation phase for the algorithm. In this phase, the bottom of the scanner was also lined with black cardboard paper as a final improvement. This ensured that even large leaves, which are not held horizontally by the plant and bend downwards, could be captured on a uniform background. This also made it possible to record video footage with a camera pointing vertically at the plants from above, for which we added a Raspberry Pi camera on top due to its ease of use and modularity. This top-view video material has not yet been used to analyze the plants. A detailed description of the technical imaging setup, including the finale image acquisition parameters, is provided in Sect. The final plant scanning platform. In this setup, the imaging geometry was standardized by rigidly fixing the camera position relative to the plant platform. An adjustable mount was used to set the camera height and angle, while the camera-to-plant distance, lens orientation, and rotational speed were all maintained fixed throughout the final validation phase. Since the primary objective of this work is disease classification rather than metric 3D reconstruction, full geometric camera calibration (e.g., intrinsic and extrinsic parameter estimation) was not necessary for the present analysis.

## Approach 1: The model for detecting the infected areas of the plants based on image segmentation

### Segmentation algorithm

The proposed algorithm for segmenting and detection is based on the frame analysis of each recording wherein the segmentation analysis is applied to each frame of the video individually. The segmentation algorithm consists of three main steps including pre-processing of the video, region of interest segmentation, and removal of miss-segmented areas followed by extraction of required statistical features. In the pre-processing step, after frame extraction and cropping, each frame is subjected to contrast adjustment and histogram equalization which are common pre-processing operations and normally intended for sharpening the gradient around the infected areas without introducing amplifying noise. After pre-processing step, the RGB frames are converted to HSV frames to separate color from strength and brightness followed by masking operation. To target the plant infected tissues, lower and upper bounds for each HSV component have been extracted wherein the lower bound is $L=(H_{low}=23.5, S_{low}=96.9, V_{low}=153)$ and the higher bound is $H=(H_{high}=33.5, S_{high}=255, V_{high}=255)$. The bounds have been found inspired from the range of HSV values of infected color tissue empirically. Finally by applying thresholding operation the areas with corresponding HSV values ranged between the low and high threshold values a binary segmented frame is generated. To further improve the segmentation result (removing the outlier or making the infected areas as connected) morphological operation is applied on the segmented frame. However, the latter operation is applied just on leaves segmentation part. More detailed description of the segmentation algorithm is depicted in Algorithm 1.

**Algorithm 1:**
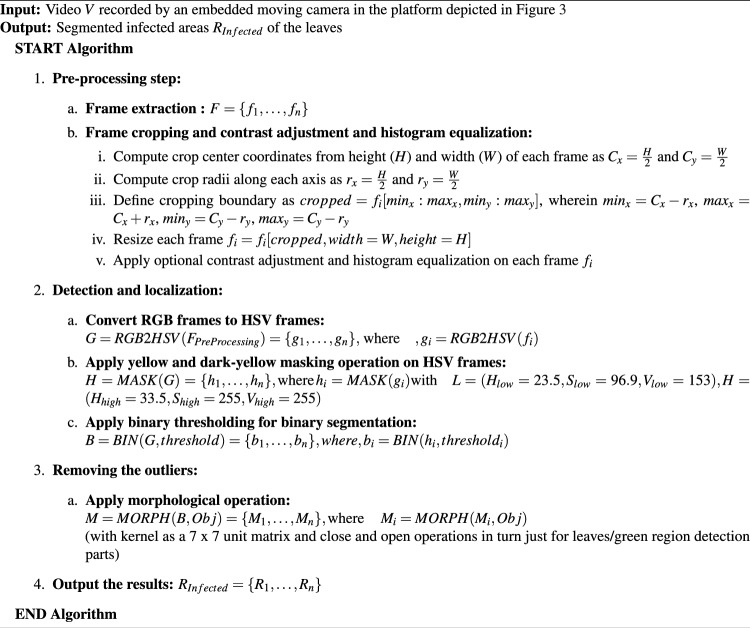
Segmenting the infected leaves.

### Candidates selection

**Fig. 4:**
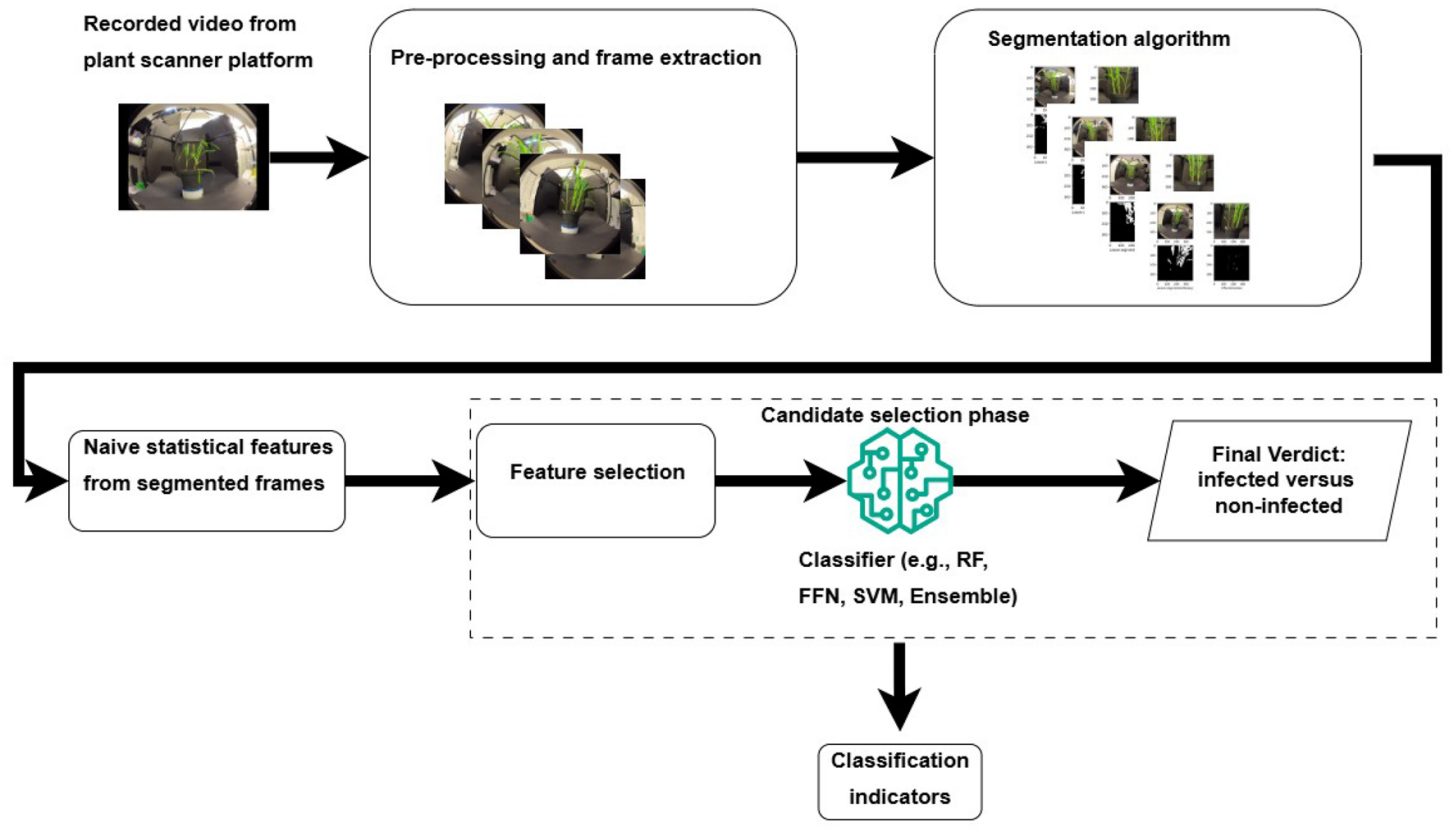
Complete pipeline of the platform (first approach).

The second phase of this segmentation approach deals with the candidate selection utilizing machine learning approach. The output of the last process of the first phase has been used in the current candidate selection phase. In this step, naive statistical features from frames set of each video are handcrafted and extracted. The extracted handcrafted features are mainly based on the ratio of detected segmented areas to the segmented plant area (detected leaves and segmented areas) of each frame. The statistical features are computed as min, max, mean, std, first and third quartile, median and mode, and the number of frames with ration higher than mean of ratios of all frames across all of the extracted frames of a plant video labeled as infected or healthy. These features are fed to three classical machine learning algorithms and one hybrid algorithm to label the plant as infected or non-infected. Several classifiers were trained to classify the plant based on the extracted features. The choices of classifiers include support vector machine (SVM), random forest (RF), and a deep feed forward network (FFN). SVM is a classifier algorithm from the family of statistical learners commonly used for classification or regression tasks. The classification task on non-linear separable data can be performed on a higher dimensional space through kernel functions. The wide popularity of the SVM is due to its relative robustness against overfitting phenomenon. RF, another classifier used in our problem, is an ensemble method introduced in 2001 based on the bagging (bootstrap aggregating) concept for both classification and classification tasks. Its resistance against overfitting and its strength in training through different data types are important characteristics of this classifier. One of the main important extensions of RF is the optimized gradient boosting decision tree^69^. Feed forward network is one of the main categories of artificial neural network (ANN) classifiers which have been established since the 60s^70^. The ANNs are non-linear models of neurons trying to mimic the functionality of biological neurons in humans. The structure of an ANN consists of neurons in which both classification and regression tasks can be performed by flowing the features inside the trained network layers. FFNs are also commonly utilized as the front end of deep learning architectures^71^. The popularity of FFNs has increased mainly due to the ability to approximate any continuous function with high accuracy indicating their universal approximation capability as well as their training capability which enables them to approximate the Bayes discriminant^71,72^. Besides the data quality, the performance of the network highly depends on the number of hidden layers and neurons, the choice of activation functions, the number of connections, the hyperparameters (e.g. learning rate) and the utilized optimization algorithm. Many algorithms that exist in the literature which carried out the FFN training. Such algorithms range from the first order to second order and are either the gradient-based or not which determines the required computational and/or memory complexity of each algorithm. Finally, an ensemble model based on the combination of classifiers has been introduced and trained for the sake of classification enhancement. Three classifiers including SVM, RF and FFN have been combined through an ensemble learning method after the training as depicted in Fig. 4. The performance of the classifiers has been measured in a stratified cross fold validation setting. The optimum utilized meta parameters of each classifier are selected on a greedy search basis. The performance of the hybrid model has been evaluated based on the average voting method in which all of the weights have been considered equally: let $v_{i}$ denote the output for each classifier in the hybrid model, then the final prediction vote of the model is computed as follows:

$$\begin{aligned} V = \frac{1}{3}(v1+v2+v3) \end{aligned}$$

It must be also pointed out that in the rare case of a tie, the label vote corresponding to the classifier with the higher validation accuracy will be selected. The description of the candidate selection algorithm is depicted as follows in Algorithm 2.

**Algorithm 2:**
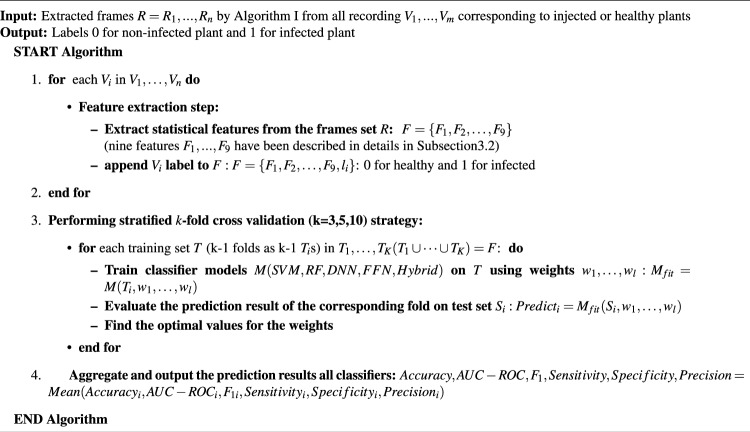
Candidate selection.

The overall detection scenario including both segmentation and candidate selection algorithms is shown in the following figure (Fig. 4).

## Approach 2: The model for detecting the infected areas of plants based on pre-trained model YOLO11

**Fig. 5:**
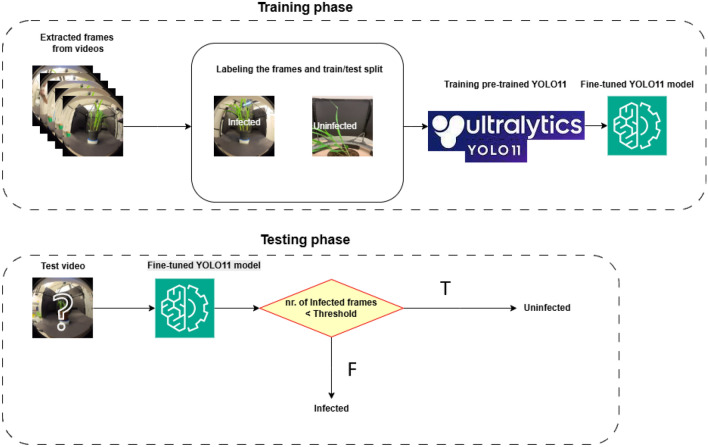
The complete pipeline of the proposed platform.

In this approach a data-driven deep learning paradigm, specifically a single-stage CNN-based detector YOLO11 directly mapping image pixels to bounding boxes and class probabilities is adapted in a detection pipeline. By leveraging transfer learning, the model learns hierarchical, task-optimized representations that overcome the limitations of handcrafted features and shallow classifiers used in the classical ML-based pipeline. Such an end-to-end learning enables complex DL-based models like YOLO11 to capture complex, variable disease symptoms, achieving near-perfect generalization (AUC 0.99–1.00). Furthermore, this pipeline is considered to be architecture-agnostic and can readily incorporate similar DL-based complicated models. In this approach, a CNN-based pre-trained model YOLO11 is used in the object detection phase to detect the infected areas in the plants. YOLO11 is almost the latest version of Ultralytics YOLO which is a pre-trained model based on a deep learning architecture utilized for object detection tasks. YOLO11 proposes some advances on previous YOLO versions in terms of architecture and training procedure which enables a wider range of object detection jobs. The YOLO11 is initially used as a pre-trained model and further fine-tuned to be adapted to our problem use-case. In fact, the YOLO11 is used for object detection in frames of videos and later on a decision based on a defined threshold is used to label the videos as infected or healthy. General pipeline of the proposed approach is shown as follows (Fig. 5).

## Results

### The final plant scanning platform

**Fig. 6:**
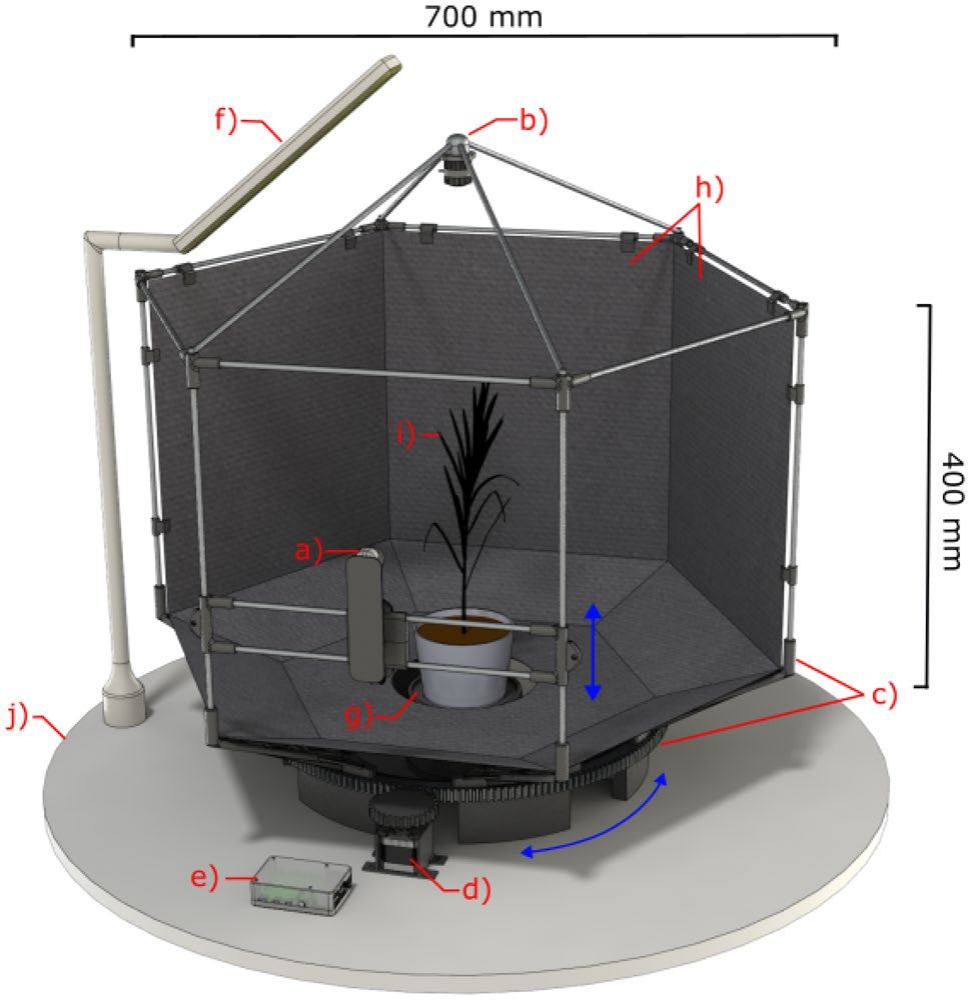
Phase Four—Final Plantscanner for 360$^{\circ }$ image capturing of the infected plant with 150 L scan volume: (**a**) side-view camera (Insta360 Go 2 ), (**b**) top-view camera (Raspberry Pi camera), (**c**) 360$^{\circ }$ camera rotation unit, (**d**) stepper motor, (**e**) control unit, (**f**) lamp, (**g**) adjustable plant platform, (**h**) high-contrast background, (**i**) scan object, (**j**) base plate.

Plants are sensitive subjects for scanning. Minor movements or vibrations cause the flexible plant material to move and, therefore, reduce the recording quality. To generate high-quality video material and consistent test environments, we built a scanning platform that allows 360$^{\circ }$ rotation of cameras around the plant (Fig. 6). The final Plantscanner system with 150 L hexagon scan volume contains light-hole aluminum round rods with a diameter of 6 mm for the struts. The connections between the rods and the camera mounts were printed with NYLON 12 POWDER using an SLS 3D printing process (Fuse 1, Formlabs GmbH, Berlin, Germany). Further final components are (Fig. 6):

- Insta360 Go 2 side-view camera (GING2XX/A, Arashi Vision Inc., Shenzhen, China) with integrated wide-angle lens and iPhone XR (MRY92ZD/A, Apple Inc., California, U.S.) with an adjustable camera mount in height and angle.
- Raspberry Pi top-view camera with stacked 12.3 megapixel Sony sensor (IMX477R, Cambridge, Great Britain) and a mounted Raspberry Pi 6 mm wide-angle lens (PT361060M3MP12).
- Camera rotation unit based on a slewing ring driven by a stepper motor (d) for vibration-free 360$^{\circ }$ camera movement around the scanned object which does not experience any movement between the series of images.
- Polulu Nema 17 size bipolar stepper motor with 200 steps per revolution.
- Control unit with Arduino Nano microcontroller (Arduino SA, Chiasso, Switzerland) and DRV8824 stepper motor controller (Texas Instruments, Dallas, Texas) for controlling rotational movement speed and Raspberry Pi 4 (Model B 2GB RAM 2018, Cambridge, Great Britain) for image capturing.
- 14 W LED light source with adjustable light intensity to ambient conditions with light color settings 3000K, 4500K, 6000K (YTZZ6SSTQGGMDN9QPM5Y-G, SerDa-Run).
- Adjustable plant platform height to exclude plant pot from scan volume.
- Non-reflecting and high-contrast black background (replaceable).

In the designing of our low-cost scanner, we focused on using only conventional, easily accessible materials. Compared to other studies^17–19,26,31^, the low-cost factor makes it possible to replicate and establish the scanner in smaller studies. In addition, the handling of the scanner is intended for anyone without in-depth training. The base plate is a conventional wooden plate that provides a secure stand and makes it easy to mount additional features, such as additional light sources. We used light-hole aluminum rods as the frame material, which are well-suited for this purpose due to their strength and lightness. All connectors and the brackets for the rotation unit are made of SLS 3D printed material like nylon. Waterproof materials such as ABS or PETG are also possible with FDM 3D printing. These parts, in combination with a ball-bearing slewing ring and a stepper motor as the drive, enable us to rotate the camera 360$^{\circ }$ with low vibration at a speed of 12 s/revolution. These settings made it possible to reduce image blurriness to a minimum. In the current setup, the plant remains stationary while the camera system rotates around it at a constant angular velocity. To acquire sharp, high-resolution image data with a large field of view, videos were recorded using an f/2.2 wide-angle lens at 2560 × 1440 pixels and 50 fps. Video exposure, ISO, and white balance were controlled automatically by the camera. This setting with fixed rotation speed, effectively minimizes motion-induced blur and allows sharp individual frames to be extracted during post-processing. A wide-angle lens further enables a large field of view while preserving image sharpness and resolution. So, the present classification pipeline does not rely on exact angular triggering of individual frames. Consequently, no hardware-level synchronization between motor steps and frame acquisition was implemented. Each plant was scanned once during a complete 360$^{\circ }$ rotational acquisition, resulting in a single video sequence containing approximately 600 images. The camera itself can be adjusted in height and angle to the scan object. During image acquisition, the camera height was adjusted individually to align the upper third of the plant with the center of the imaging area. In this setup, a dedicated LED lamp operated at fixed color temprature of 4500 K, is employed to mitigate shadows arising from overlapping leaf structures, while also preventing overexposure, insufficient illumination, and non uniform light distribution conditions that would otherwise lead to loss of interpretable image information. Combined with fixed camera parameters and a black background, this illumination configuration ensures consistent and standardized lighting, thereby yielding stable color intensity for the subsequent analysis pipeline which is particularly important for reproducible detection of disease-associated color changes such as chlorosis and necrosis. Furthermore, should the need arise, the LED intensity and color temperature, as well as the camera exposure settings, can be adjusted prior to image acquisition. In addition, it enables consistent illumination and, therefore, consistent color intensity for the further algorithm. The currently used 45$^{\circ }$ offset to the lens can be freely adjusted. The non-reflecting and high-contrast black background was designed from cost-efficient cardboard paper. The black background ensures that only the plant is considered in the later analysis. Furthermore, the strategy of rotating camera setup allows the same species to be observed from multiple viewpoints, thereby exposing regions that may be hidden in one view but visible in another. Importantly, because the plant remains fixed and only the camera rotates, the setup avoids additional motion-related deformation and minimizes the likelihood of persistent view-dependent occlusion across the full video sequence. The scanner system created in this manner therefore, allows for cost-effective and rapid capture of smaller plants in a uniform and consistent test environment.

### Generated database

**Fig. 7:**
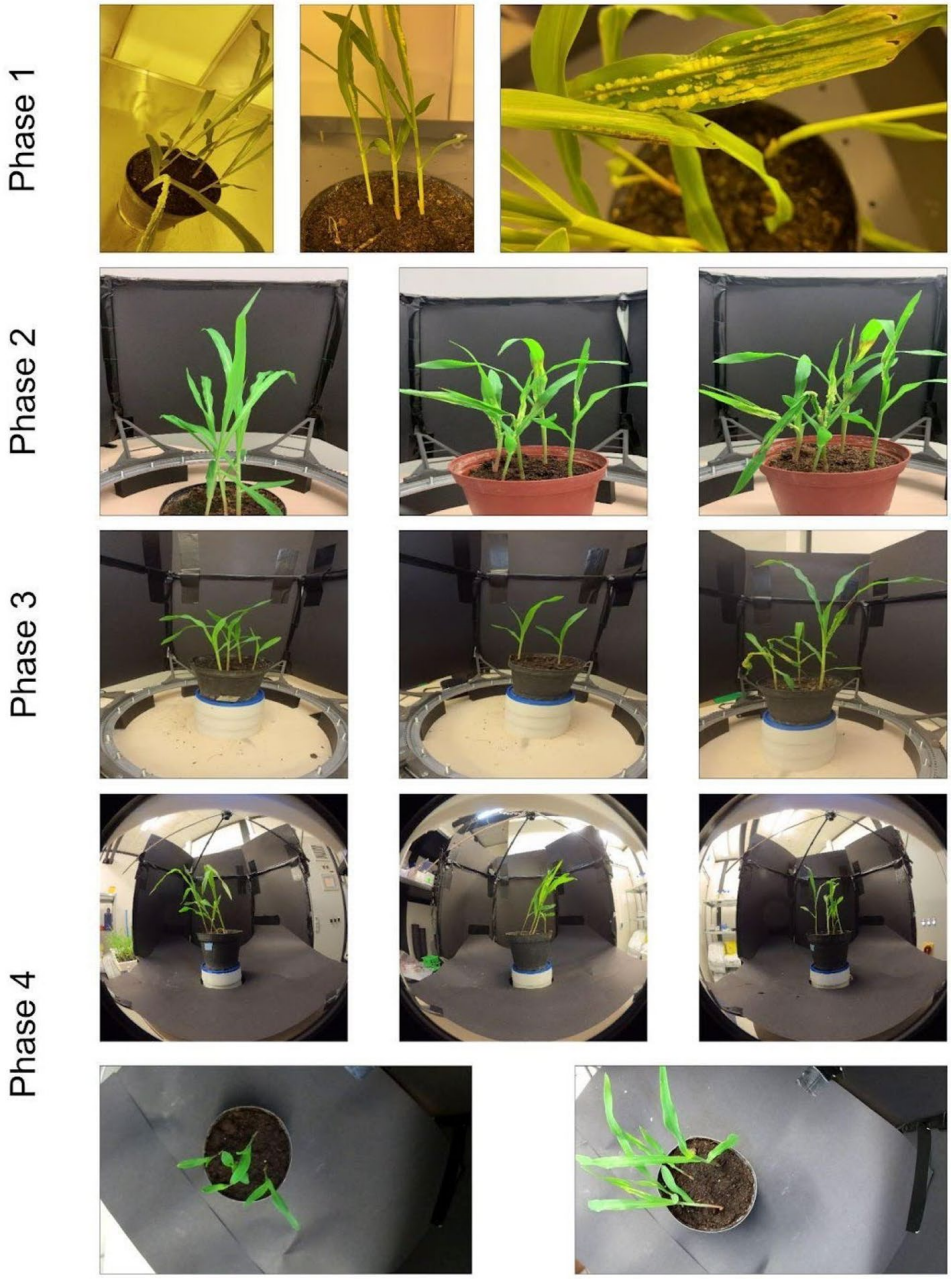
Example of recordings during the development phases: Images shown are sample images of maize plants from the four stages of development. The technical details are described in Sect. "Materials and methods".

During the further development of the scanning platform and the algorithm, insight was gained that led to improvements in the recording as well as the detection process. As a result, in the four development phases, a total of 2090 pictures and 2238 videos were generated. In phase one, pictures of plants were taken with private mobile phones. The aim was to visualize infected areas clearly. To generate defined parameters for recording, an iPhone 10 was used as a camera in Phase 2, as shown in Fig. 2. For Phase 3, a more vibration-resistant camera was used. To deal with bigger plants, the camera was moved further away from the scanned object, and an external light was installed. In Phase 4, the last background optimization was done. Samples of the generated data are shown in Fig. 7. This crafted dataset was intentionally designed not only to support the present proof-of-concept study, but also to serve as a reusable resource for the scientific community in future investigations. It is worth mentioning that, the methodology is not inherently limited to this dataset and since the classification and detection components are based on a data-driven learning strategy, the same framework can be applied to real-life datasets. The summary of generated image and video material is presented in Table 1 below. The reported numbers correspond to acquired images or videos rather than unique plant individuals, as individual plants were in some cases recorded repeatedly at different stages of symptom development.

**Table 1:** Generated picture/video materials during development phases: The numbers reported for healthy and infected conditions refer to acquired image/video recordings and not to unique plant individuals.

**Phase**	**Camera system**	**Camera position**	**Material gained**	**Plant condition**	
				**healthy**	**infected**
One	Cell phones from different manufacturers and of different quality	random	2090 pictures	random collection of healthy and infected plants	
Two	iPhone 10	sideview	88 videos	39	49
Three	ActionCam	sideview	1893 videos	200	1693
Four	ActionCam	sideview	157 videos	79	78
	RaspiCam	topview	100 videos	50	50

Individual plants may have contributed multiple recordings acquired at different time points during symptom development.

### Performance measurement

To evaluate the classification models presented in this work, the elements of the extracted confusion matrix including true positive (TP), true negative (TN), false positive (FP), and false negative (FN) values will be considered. TP indicates the truly validated infected plants correctly predicted by the prediction method, and TN represents the healthy plants correctly predicted. FP denotes the healthy plants incorrectly predicted as the infected ones and finally, FN indicates the experimentally validated infected plants incorrectly predicted as the healthy plants. The classification performance is evaluated by accuracy (ACC), sensitivity (SN), specificity (SP), and precision (PR) indicators. Furthermore, the receiver operating characteristic (ROC) curve representing the trade-off between the TP rate or sensitivity and the FP rate or 1-specificity has been extracted. The definition of the aforementioned classification parameters is as follows:

*Accuracy* It is the percentage of the correct predictions calculated below.

1 $$\begin{aligned} Accuracy = \frac{TP + TN}{TP + FP + TN + FN} \end{aligned}$$

*Sensitivity (Recall)* It indicates the percentage of infected plants sites that have been predicted correctly.

2 $$\begin{aligned} Sensitivity = \frac{TP}{TP + FN} \end{aligned}$$

*Specificity* It shows the percentage of non-infected (healthy) sites that have been predicted correctly as non-infected.

3 $$\begin{aligned} Specificity = \frac{TN}{TN + FP} \end{aligned}$$

*Precision* It indicates the positive predictive value and is calculated as follows.

4 $$\begin{aligned} Precision = \frac{TP}{TP + FP} \end{aligned}$$

*F1-score* The F1-score calculates a score based on the combination of precision and recall by computing the harmonic mean of those metrics as follows.

5 $$\begin{aligned} F1 = 2 \frac{Precision.Recall}{Precision + Recall} \end{aligned}$$

### Models evaluation

In this section the experimental results corresponding to two introduced approaches are presented. In the first approach, the algorithms in the classification models have been trained by handcrafted features from frames extracted from 28 balanced videos (14 infected versus 14 healthy plants) dataset and the models have been frozen by evaluation using k-fold cross-validation setting for k=3,5,10. The evaluation on the test set has been presented by calculating the mean of all classification parameters achieved per each fold. Although there are nine statistical features (mean, standard of deviation, min, max, the first quartile, the third quartile, median, mode and the number of frames whose infected ratio is bigger than the mean of infected ratios of all frames) have been calculated from the segmentation algorithm in the first phase, a subset of the features is used for the candidate selection phase. All of the features have been calculated from the pixel values of the infected areas of the extracted frames. For example, to calculate the first feature i.e., mean for one recording, the ratio of the infected area of each extracted frame to the whole area of the plant is calculated and then the mean is taken over all of these ratios across all frames. The same process is performed for other features as well. To select the best features, a feature selection algorithm is used. According to the feature selection algorithm, the following features have been found to present the best classification performance among other combinations of the features: (1) Max, (2) The first and third quartile, (3) Mode, and (4) Number of frames whose infected ratio is bigger than the mean of infected ratios of all frames. The used classical SVM model utilizes linear kernel. The proposed Random Forest model uses 200 decision trees with maximum depth of 90 and minimum number of splitting samples of 10 in bootstrapping mode. Corresponding FFN model consists of 3 layers and the parameters are shown in Table 2 as follows. According to the evaluation of the trained models, in the first approach, the best performance is achieved by the SVM model in a 10-fold stratified CV training scenario wherein the accuracy of 0.90 can be achieved.

**Table 2:** Settings of the proposed deep FFN architecture.

Sequential neurons	(64, 32, 1)
Activation function	(ReLU, ReLU, Sigmoid)
Optimizing function	adam
Loss function	binary cross entropy
Epochs	100
Batch size	16

**Fig. 8:**
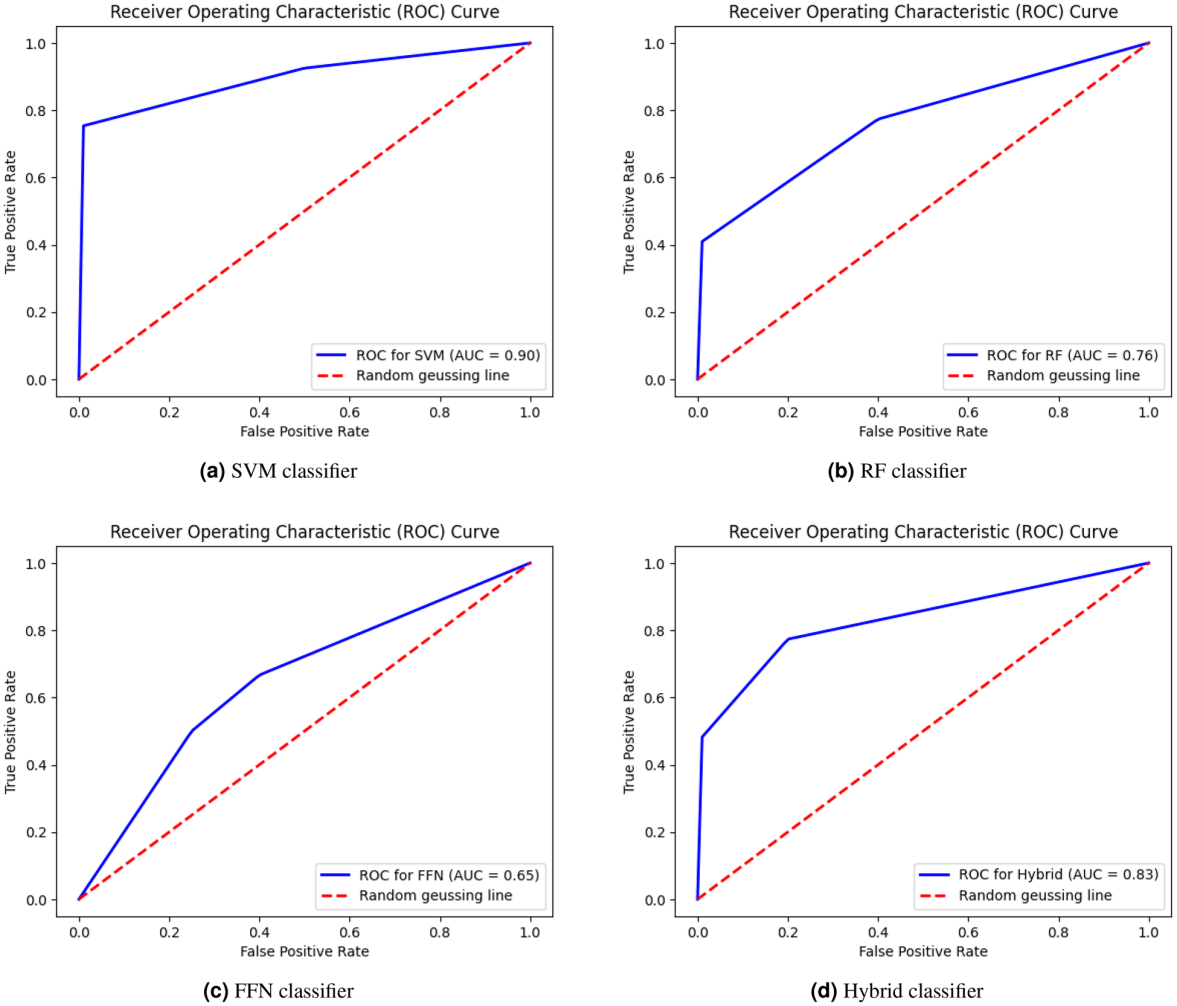
ROC of four different classifiers corresponding to the best fold.

To experimentally evaluate the second approach, at first the YOLO11 pretrained model has been trained using frames extracted from the training dataset. To reduce the training cost, we have extracted just about $10\%$ of the frames (equally distributed) of each video of the set. Fine-tuning included adjusting learning rates (0.01), batch size (32), and epochs (50), with no augmentation to preserve biological relevance. The model has been trained to distinguish the infected frames from non-infected frames in real-time. The fine-tuned model based on this frames set is then utilized to label the extracted frames. The model contains 28.358.626 learning parameters corresponding to the same amount of gradient values through 176 layers with a learning rate of 0.01. The image frames are of size 224 × 224 pixels. Each epoch iteration takes about 1.1 seconds on average on a Tesla T4 GPU platform using 57 MB in an optimized weights setting. So, since the target objective is based on detecting the infected plants according to the visual symptoms, the labeling criteria for infected/uninfe-/cted (healthy) samples are completely upon appearance of visual symptoms in the plant subjected to the infection. Also, we have reported the results from the collected phases (two to four) utilized in the training stage as they represent the results corresponding to different settings tolerating only marginal modifications. It means that the utilized images in training, validating and testing are collected from phases two to four. Since the generated dataset across phases, contains multiple image frames (extracted from videos) from the same individual plant specimen, we carried out splitting task at the plant group level (infected versus healthy or uninfected) rather than at the image frame level. All frames derived from that plant were allocated either entirely to the training set or entirely to the validation and test sets. In the training process of the proposed pipeline, a dedicated balance test set consisting of 458 frames from 10 videos (5 videos per class) was separated at the process outset and remained completely untouched and inaccessible during model development, parameter tuning, and finalization. The training has been performed using a balanced train set of frames consisting of 2800 frames (2400 frames for train set and 400 frames for validation set) extracted from a separate set of videos. The final model shows an excellent validation accuracy and AUC-ROC of 0.9998. Summary of the results corresponding to the first approach and the second approach are presented in Table 3 which is followed by the ROCs and corresponding AUC individually in Fig. 8 as follows. It is worth mentioning that the results corresponding to the best fold in the first approach with the highest accuracy for each classifier is depicted in the table and plots.

**Table 3:** Classification parameters achieved through cross-fold validation for$k=3,5,10$using four different classifiers.

**Model**	**Accuracy**	**Sensitivity**	**Specificity**	**Precision**	**F1-score**	**AUC-ROC**
SVM-k=10	0.90	0.85	0.95	0.85	0.73	0.90
FFN-k=3	0.78	0.73	0.83	0.83	0.77	0.65
RF-k=3	0.76	0.73	0.78	0.89	0.73	0.76
Hybrid-k=3	0.83	0.73	0.93	0.93	0.79	0.83
Approach 2	0.99	0.99	0.99	0.98	0.99	0.99

**Fig. 9:**
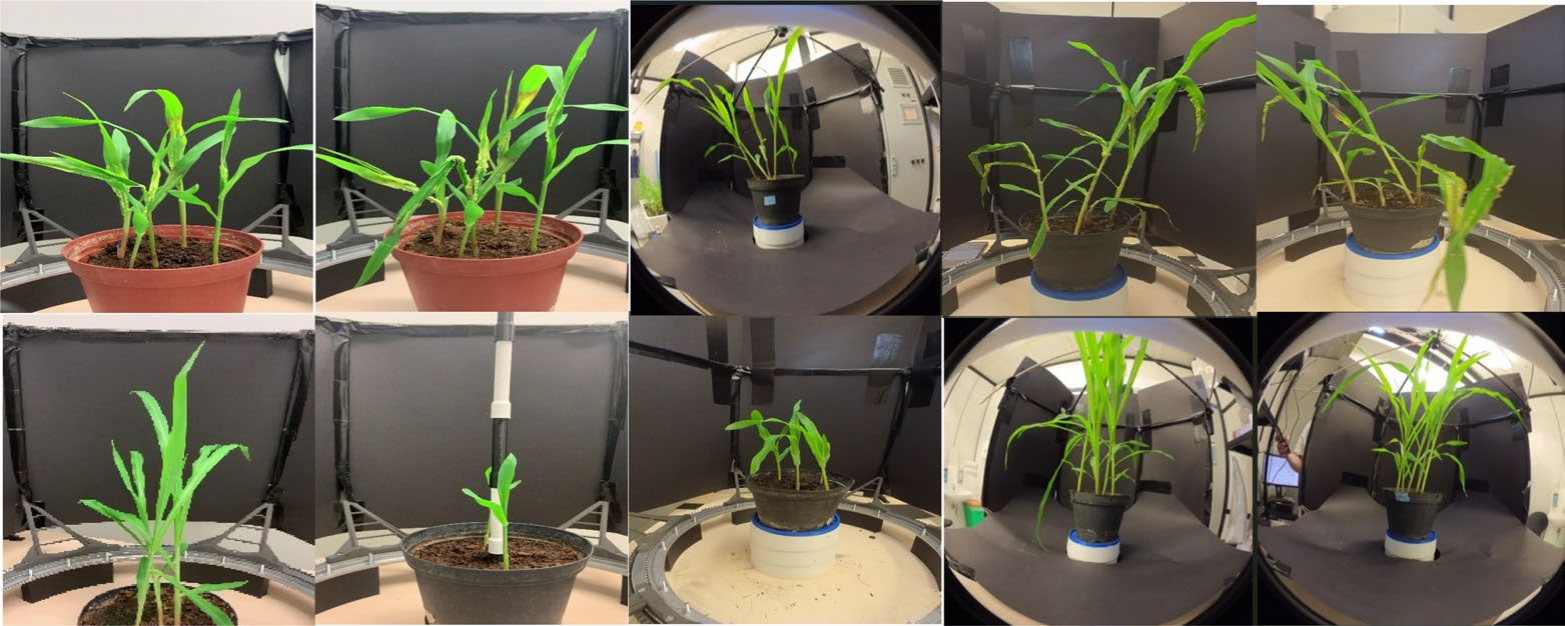
Test samples set: first row (top) shows five infected frames and the second row shows five uninfected frames.

According to the results shown in the Table 3, the second presented approach shows an excellent performance with accuracy 0.99 as expected. This indicates the superiority of a complex deep learning-based data-driven model on traditional models. However each approach brings its corresponding advantages concerning the computational cost, interpretability, utilized resources, and demanding performance which can be decided as per use-case in practice. Also, the detailed results corresponding to the pipeline of superior approach (approach 2) on the test (frames of 10 balanced videos) are shown in the following table (Table 4) followed by representative examples of the test set in Fig. 9.

**Table 4:** Detailed results of the proposed pipeline on the test set.

**Test samples**	**Data generation phase**	**Detected frames as infected (ratio)**	**Label**	**Final verdict**
Sample-1	phase 2	$100\%$	Infected	Infected
Sample-2	phase 2	$100\%$	Infected	Infected
Sample-3	phase 4	$45\%$	Infected	Infected
Sample-4	phase 3	$100\%$	Infected	Infected
Sample-5	phase 3	$100\%$	Infected	Infected
Sample-6	phase 2	$0\%$	Uninfected	Uninfected
Sample-7	phase 2	$5\%$	Uninfected	Uninfected
Sample-8	phase 3	$0\%$	Uninfected	Uninfected
Sample-9	phase 4	$0\%$	Uninfected	Uninfected
Sample-10	phase 4	$0\%$	Uninfected	Uninfected

**Fig. 10:**
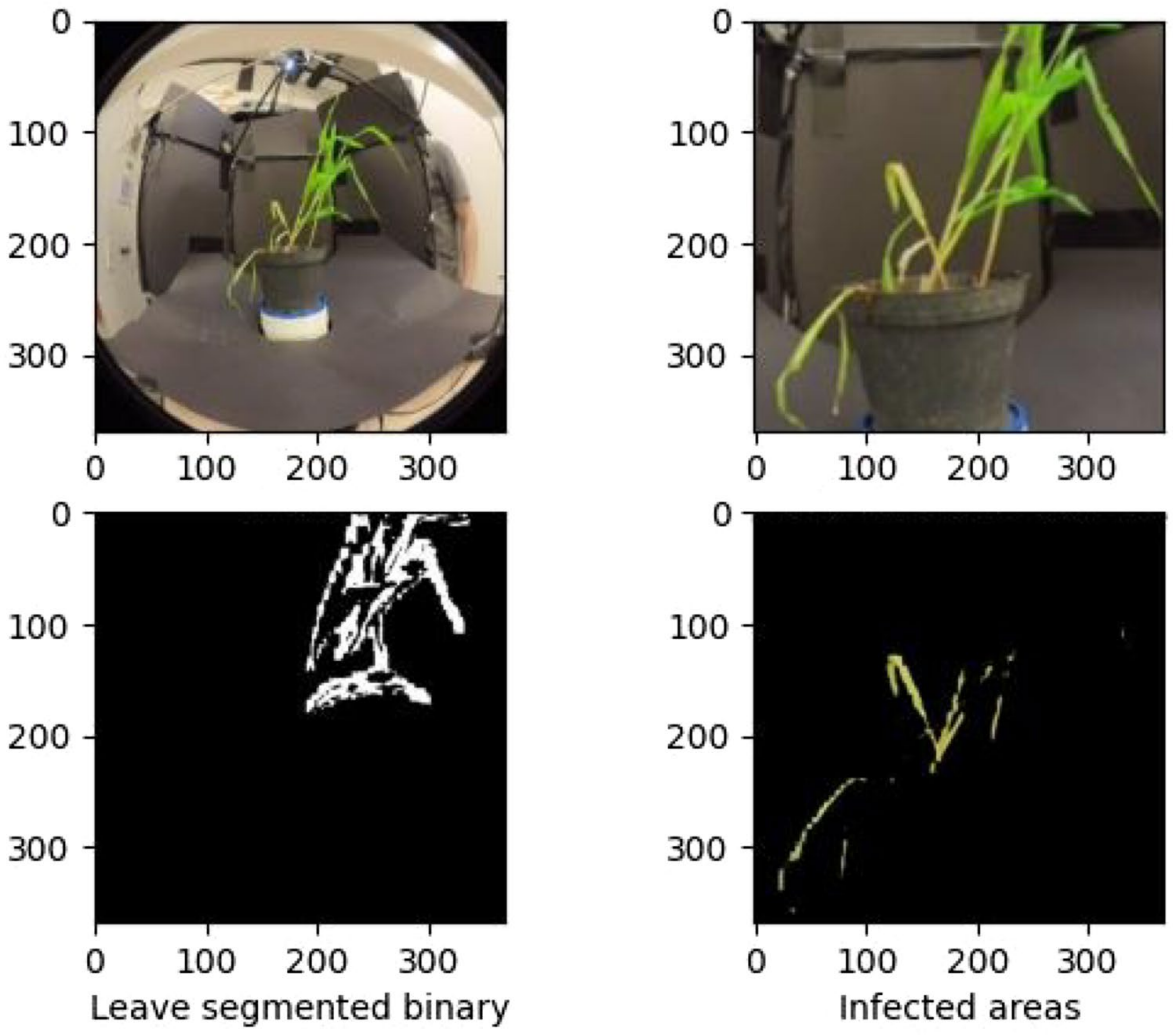
Infected areas by the first approach based on computer vision.

**Fig. 11:**
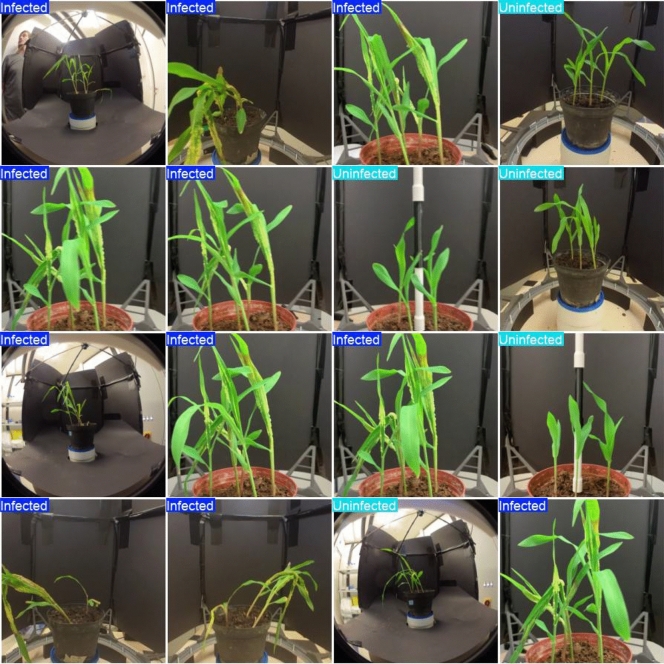
Detection results output from the second approach based on YOLO11 model.

As shown in Table 4, the frame-level predictions are highly consistent with the ground-truth plant labels across the test set. In the absence of a straightforward analytical formulation for aggregating frame-level decisions into plant-level predictions, a threshold of 40$\%$ was initially selected empirically and subsequently validated on the test set. The obtained results support the robustness of this choice, as no uninfected plant produced more than 10$\%$ infected-predicted frames, providing a clear margin from the decision threshold and reducing the risk of erroneous plant-level classification. According to the result on the test set, only one sample exhibited a proportion of counter-class predictions exceeding the selected threshold, with 55$\%$ of its frames assigned to the opposite class (uninfected). Consequently, the adopted threshold offers a practical and reliable decision criterion for the proposed framework. However, it should be noted that the threshold is application-dependent and may be adjusted according to the desired operating scenario. Lower threshold values can be adopted in more conservative screening scenarios to increase sensitivity and reduce the likelihood of missed infections, whereas higher thresholds may be preferred when a more stringent decision criterion is required. Intermediate results corresponding to both approach models are shown in Figs. 10 and 11 as follows.

**Fig. 12:**
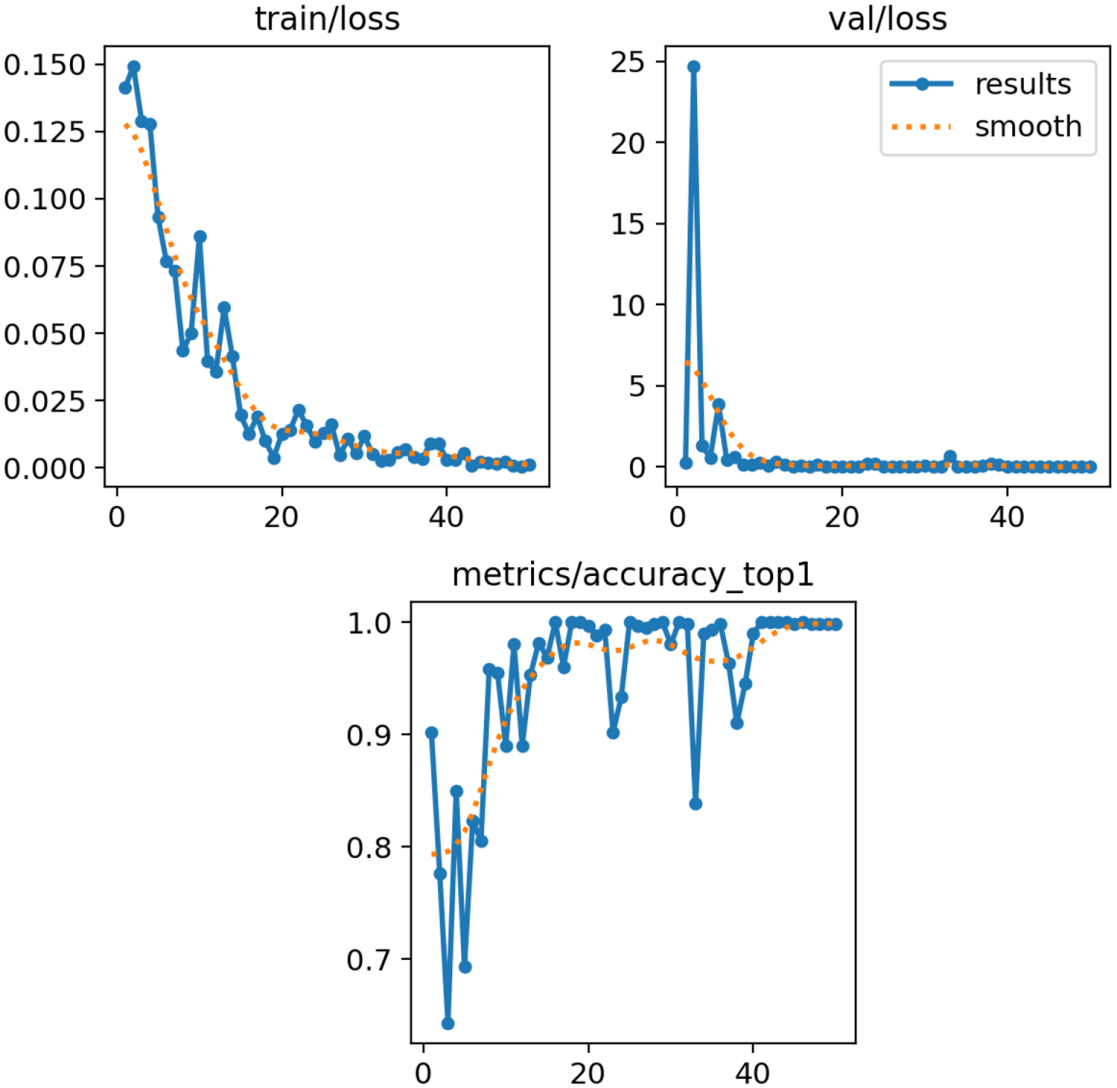
Loss/Accuracy trend of the second approach: x-axis denotes the epochs and y-axis (above) denotes the loss value and y-axis (below) denotes the accuracy value.

Also, the results corresponding to the train/loss trend as well as achieved accuracy in the second approach is shown in Fig. 12 as follows.

To contextualize the performance of the proposed YOLO11-based pipeline, we benchmarked it against two representative deep learning baselines, YOLOv8 and a ViT, each fine-tuned on the same extracted-frame dataset used for YOLO11 (Table 5). YOLOv8 is an immediate predecessor of YOLO11 within the Ultralytics YOLO family. Like YOLO11, it is a single-stage, CNN-based detector that predicts object locations and class scores in a single forward pass. Because the two share the same detection paradigm and were fine-tuned on the same data, such a comparison indicates whether the architectural and training refinements introduced in YOLO11 translate into measurable gains on this task. The Vision Transformer^73^ represents a fundamentally different design philosophy. Instead of convolutional filters, ViT divides each image into fixed-size patches, embeds them as a sequence of tokens, and processes them with self-attention layers that model global relationships across the entire image. Including ViT tests the task against the transformer paradigm, which has become a leading alternative to CNNs in recent plant disease detection work^61–65,74^. Because all three models were fine-tuned on the same dataset, the gain over YOLOv8 suggests that, on this specific tumor-symptom task, the newer version offers a practical advantage over its predecessor within the same detection family. ViT’s lower accuracy at a comparable AUC-ROC is in line with the generally greater data requirements of transformer models, which may place them at a disadvantage on a dataset of this size relative to the convolutional inductive bias of the YOLO family.

**Table 5:** Comparison results with ViT and earlier version of YOLO.

**Model**	**Accuracy**	**Sensitivity**	**Specificity**	**Precision**	**F1-score**	**AUC-ROC**
YOLO8	0.92	0.92	0.99	0.93	0.92	0.99
ViT	0.78	0.82	0.77	0.66	0.73	0.93

### Implementation details

The implementation of the proposed approaches has been done with Python version 3.12 leveraging its robust ecosystem of libraries tailored for machine learning and computer vision tasks. The primary frameworks used include Pandas, Numpy, Scikitlearn, tensorflow, and OpenCV. For implementing the second approach, package ultralytics has been imported in order to automate the whole pipeline.

## Discussion

**Fig. 13:**
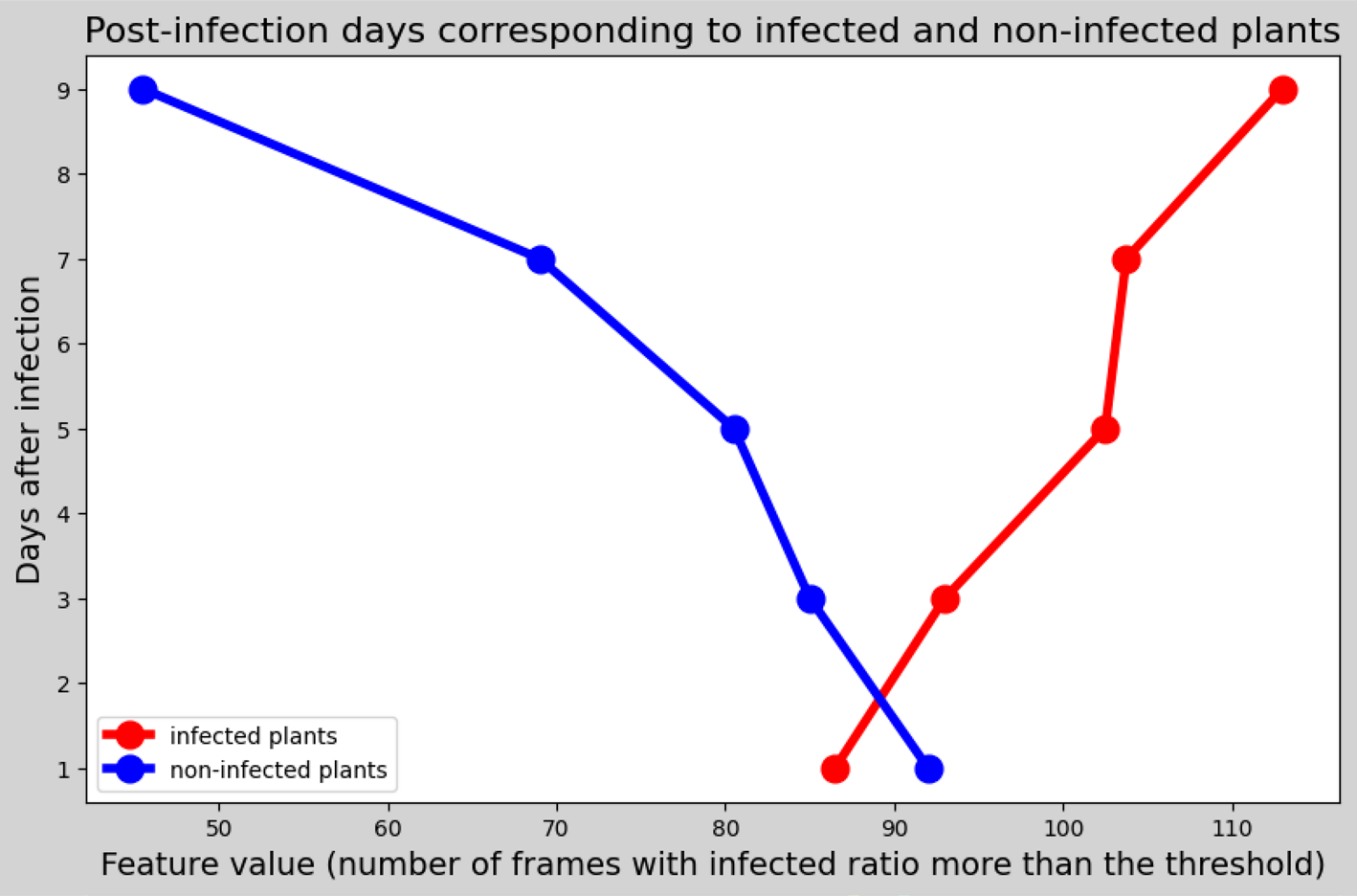
Comparison of detected frames with the ratio of the detected pixels bigger than the specified threshold value between the infected and healthy plants.

This study presents a platform for detecting tumor-based symptoms in corn caused by the pathogenic fungus *Ustilago maydis* under laboratory conditions. The *Ustilago maydis*-*Zea mays* pathosystem is an attractive system to study pathogens^75–77^. Much of our understanding of *U. maydis* infection behavior comes from genetic manipulations of the fungus, and a comparison of resulting mutants to wildtype strains in infection assays. While the symptoms in the wild differ greatly from the symptoms developed in this infection assay (see Fig. 1), differences in symptoms achieved during this assay, provide insights into the gene’s potential role in the fungus. Homology studies further allow extrapolation of these findings to other fungal pathogens, helping to identify common mechanisms of infection. This knowledge can contribute to the development of targeted strategies to mitigate pathogen infestations in crops through precise treatments. The problem with these infection assays is that the symptoms are read out manually by the respective scientists, which can lead to subjective biases. Furthermore, in order to make the workload manageable, publications often only show a few pictures of the symptoms of hundreds of plants, which is accepted in the community as standard^75–77^. To solve these problems, we have generated a platform and an algorithm that visualizes the infected plants and can then distinguish between infected and non-infected plants. The developed scanning platform was used to generate video material of the plants. The aim was to generate a modular platform that can be used by any trained person in laboratory environments, greenhouses, or other premises due to its high adaptability. The open-source platform allows for modifications to the camera systems, orientation, rotation speed, backgrounds, and lighting, making it highly customizable. To its flexible camera settings, users can scan a large number of plants in an adequate amount of time. To achieve this goal, we have placed great emphasis on designing the platform to be cost-efficient. Unlike commercial systems (e.g., WIWAM, 20,000€), our open-design scanner reduces costs by 95%, enabling wider adoption. With a production value of less than 1000€ (inclusive camera, that made the biggest position), the device opens the possibility for stable plant recordings to a wide range of users with different needs. Because existing infection detection systems do not apply to tumor-based plant pathogens, we have created our own data collection with video and images. During the different development phases we ended up with more than 2000 pictures and 2000 videos of infected and non-infected maize plants. This database was created under controlled laboratory conditions, providing video/image data that accurately represents infection stages in maize plants. To clearly observe the stages, the plants were monitored for a duration of up to 9 days, during which the infection became more visible. In the future, this database may help to develop further approaches for detecting fungal infections on plants. Using an RGB imaging system combined with the self-made scanning device, we were able to achieve a classification accuracy of over 0.99. This point will clearly discriminate between the infected plants from the non-infected ones. In Fig. 13, one of the selected features (number of frames whose infected ratio is bigger than the mean of infected ratios of all frames) has been plotted for two samples of recordings corresponding to the infected and non-infected plants.

As depicted in the above plot (Fig. 13), the number of frames whose infected ratio is bigger than the threshold corresponding to an infected plant in the 1, 3, 5, 7, and 9 days after infection is increasing, while the similar feature corresponding to a non- infected one has an opposite trend as expected. The decrease in non-infected ratio observed is due to gradual enlarging the leaves area within days without widening the infected regions since the plants are not injected. However in the first day after infection, as the symptoms have not significantly appeared on the leaves, the segmentation algorithm was not such successful in detecting the infected areas. Therefore, the difference between the frame numbers between the plants (infected versus healthy) is expected to be tiny and mainly is data-subjective. Similar plots might be expected when extracted for the other features, however it is not a must. This is an indication of the synergy between the segmentation algorithm and the infection process, showing an empirical justification of the consistency of the algorithm. Concerning to the proposed approaches and performance results, it appears as expected that YOLO11-based approach outperforms significantly in comparison to classical ML-based models used in the first approach. By this comparison, we aimed to demonstrate not merely that YOLO11 achieves superior accuracy, but also to characterize the magnitude of improvement and the conditions under which a more complex, data-driven model is justified. Through this traditional comparison, by comparing a fully classical pipeline (including a naïve segmentation step) with a state-of-the-art deep learning-based AI data driven model, a realistic and practically informative baseline for researchers considering deployment in resource-constrained or interpretability-focused settings is provided. However, it is worth mentioning that in many precise agricultural applications, classical computer vision and classical ML pipelines remain demanding due to their transparency, lighter computational requirements, and interpretability of intermediate steps. In summary, our system provides a strong basis for further work on scanning systems on plants. The device we have generated can help to develop other devices that can also be used to scan other types of plants. Furthermore, the complete developed package offers the possibility to easily share video and image material of maize plants infected with *U. maydis* among different scientists. It is possible to save the obtained image and video material and to review it long after the experiment was conducted.

## Conclusions

In this paper, we present a proof-of-concept platform for detecting tumor-based symptoms in maize leaves caused by pathogenic fungi. The prototype integrates various camera systems to capture comprehensive recordings of the plant. These videos are processed by an infection detection algorithm, which operates in two consecutive phases to identify infected plants. The platform achieves almost perfect accuracy of approximately 0.999, with an AUC-ROC of 0.999 utilizing a presented approach based on the deep learning data driven pre-trained CNN-based model YOLO11. Beyond the achieved performance, a potential strength of this pipeline lies in its model-agnostic flexibility wherein the detection module can be readily replaced with alternative data-driven architectures–including ViT-based detectors for example. This feature ensures that the pipeline can incorporate future advancements in object detection or segmentation, therefore offering an adaptable solution for plant disease monitoring in controlled studies. In addition to enhancing the detection algorithm, the platform generates a versatile dataset using different hardware configurations, providing a valuable resource for researchers. The platform is highly adaptable, cost-efficient, and capable of integrating multiple camera systems, making it suitable for diverse plant species and environments. It is worth mentioning that the proposed work validates the method only under controlled laboratory conditions on a single pathosystem and cannot yet claim universal generalization to all other samples without further training and external testing. Therefore, the platform provides a transferable framework for other plant disease samples, while its actual performance on new species or pathogens will depend on dataset-specific retraining and independent validation and has to be established and proven empirically. However such an extension is not free of challenges and some problems like visual similarity between new disease symptoms, species-dependent morphology, and domain shift or catastrophic forgetting have to be resolved just in case. Future developments include embedding the detection algorithm directly into the camera system for industrial applications, creating a mobile app version, and advancing the algorithm to assess infection stages. To address the limitations of the current work, future work will test the platform in field conditions to assess robustness against environmental variability as well. For example adjusting and elaborating synchronization, by incorporation of existing Arduino/Raspberry Pi control unit to trigger camera acquisition at predefined angular increments to achieve precise frame-to-angle correspondence could be considered. Plans also involve incorporating elaborated cutting-edge techniques like Vision Transformers^74^ to further improve detection accuracy and performance while extending to other samples or species.

## Data Availability

The generated database are provided as some frame samples in https://github.com/aTabaIMI/Plant-Scanner-THM-Marburg-MaizeLeaf with full data availability upon reasonable request.
